# Speech and neuroimaging effects following HiCommunication: a randomized controlled group intervention trial in Parkinson’s disease

**DOI:** 10.1093/braincomms/fcae235

**Published:** 2024-07-12

**Authors:** Hanna Steurer, Franziska Albrecht, Joakim Körner Gustafsson, Adeel Razi, Erika Franzén, Ellika Schalling

**Affiliations:** Department of Clinical Science, Intervention and Technology, Division of Speech and Language Pathology, Karolinska Institutet, 141 86 Huddinge, Stockholm, Sweden; Research & Development Unit, Stockholms Sjukhem, 112 19 Stockholm, Sweden; Division of Physiotherapy, Department of Neurobiology, Care Sciences and Society, Karolinska Institutet, 141 83 Huddinge, Stockholm, Sweden; Women’s Health and Allied Health Professionals Theme, Medical Unit Occupational Therapy & Physiotherapy, Karolinska University Hospital, 141 57 Huddinge, Stockholm, Sweden; Department of Clinical Science, Intervention and Technology, Division of Speech and Language Pathology, Karolinska Institutet, 141 86 Huddinge, Stockholm, Sweden; School of Psychological Sciences, Turner Institute for Brain and Mental Health, Monash University, Clayton, VIC, 3800, Australia; Wellcome Trust Centre for Neuroimaging, Institute of Neurology, University College London, London WC1N 3AR, UK; CIFAR Azrieli Global Scholars Program, CIFAR, Toronto, ON M5G 1M1, Canada; Research & Development Unit, Stockholms Sjukhem, 112 19 Stockholm, Sweden; Division of Physiotherapy, Department of Neurobiology, Care Sciences and Society, Karolinska Institutet, 141 83 Huddinge, Stockholm, Sweden; Women’s Health and Allied Health Professionals Theme, Medical Unit Occupational Therapy & Physiotherapy, Karolinska University Hospital, 141 57 Huddinge, Stockholm, Sweden; Department of Clinical Science, Intervention and Technology, Division of Speech and Language Pathology, Karolinska Institutet, 141 86 Huddinge, Stockholm, Sweden; Department of Public Health and Caring Sciences, Speech-Language Pathology, Uppsala University, 751 22 Uppsala, Sweden

**Keywords:** resting-state functional MRI, effective connectivity, acoustic analysis, voice sound level, Acoustic Voice Quality Index

## Abstract

Speech, voice and communication changes are common in Parkinson's disease. HiCommunication is a novel group intervention for speech and communication in Parkinson’s disease based on principles driving neuroplasticity. In a randomized controlled trial, 95 participants with Parkinson’s disease were allocated to HiCommunication or an active control intervention. Acoustic analysis was performed pre-, post- and six months after intervention. Intention-to-treat analyses with missing values imputed in linear multilevel models and complimentary per-protocol analyses were performed. The proportion of participants with a clinically relevant increase in the primary outcome measure of voice sound level was calculated. Resting-state functional MRI was performed pre- and post-intervention. Spectral dynamic causal modelling and the parametric empirical Bayes methods were applied to resting-state functional MRI data to describe effective connectivity changes in a speech-motor-related network of brain regions. From pre- to post-intervention, there were significant group-by-time interaction effects for the measures voice sound level in text reading (unstandardized *b* = 2.3, *P* = 0.003), voice sound level in monologue (unstandardized *b* = 2.1, *P* = 0.009), Acoustic Voice Quality Index (unstandardized *b* = *−*0.5, *P* = 0.016) and Harmonics-to-Noise Ratio (unstandardized *b* = 1.3, *P* = 0.014) post-intervention. For 59% of the participants, the increase in voice sound level after HiCommunication was clinically relevant. There were no sustained effects at the six-month follow-up. In the effective connectivity analysis, there was a significant decrease in inhibitory self-connectivity in the left supplementary motor area and increased connectivity from the right supplementary motor area to the left paracentral gyrus after HiCommunication compared to after the active control intervention. In conclusion, the HiCommunication intervention showed promising effects on voice sound level and voice quality in people with Parkinson’s disease, motivating investigations of barriers and facilitators for implementation of the intervention in healthcare settings. Resting-state brain effective connectivity was altered following the intervention in areas implicated, possibly due to reorganization in brain networks.

## Teaser

Steurer *et al*. conducted a randomized controlled trial to investigate speech and neuroimaging effects following HiCommunication, a group intervention for speech and communication in Parkinson’s disease. Clinically relevant effects on voice sound level, positive effects on measures of voice quality as well as changes in effective brain connectivity were found.

## Graphical Abstract

**Graphical Abstract:**
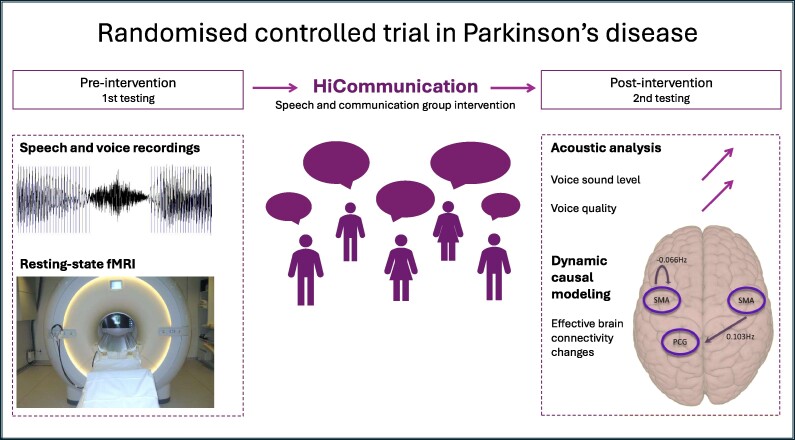

## Introduction

Speech, voice and communication changes are found in up to 90% of people with Parkinson’s disease.^1,2^ Hypokinetic dysarthria is the motor speech disorder most often associated with Parkinson’s disease. Neuromuscular changes caused by the disease affect multiple aspects of speech production, resulting in impairment of respiration, phonation, articulation, resonance and prosody.^3^ Furthermore, difficulties monitoring and scaling up the effort required to produce sufficient speech loudness are common in Parkinson’s disease.^4^

Pharmacological and surgical approaches for managing Parkinson’s disease generally alleviate motor symptoms but their impact on speech and voice function may not be equally beneficial.^5-7^ However, intensive behavioural interventions specifically aimed at improving speech and voice have demonstrated favourable effects on communication. The Lee Silverman Voice Treatment (LSVT-LOUD®) is by far the most studied intervention for speech and voice in Parkinson’s disease showing effects up to two years post-treatment.^7-11^ However, transfer of the changed speech behaviour, i.e. generalization of increased vocal loudness to other situations than during clinical treatment, remains a challenge.^3^

HiCommunication is a novel intervention for speech and communication in Parkinson’s disease, focusing on improving speech and voice technique by practising using a louder voice with a good voice quality and clear speech. The intervention is based on principles driving neuroplasticity and aims to address challenges with retention of changes following speech and voice intervention as well as transfer to settings outside the clinic. In a pilot study, high compliance and acceptability of HiCommunication have been shown.^12^ The HiCommunication intervention has been investigated in the EXercise in PArkinson’s disease and Neuroplasticity (EXPANd) trial; a randomized controlled trial (RCT) including 95 participants with Parkinson’s disease aimed at studying the link between behaviour changes following intensive intervention and structural and functional brain changes (ClincalTrials.gov: NCT03213873, for details, see study protocol^13^ and OSF page (https://osf.io/s952g/). Within the EXPANd trial, positive results for the main outcome measure of HiCommunication, voice sound level in text reading, have been shown.^14^

The evidence for neuroplastic changes following intensive speech and voice intervention in Parkinson’s disease is still limited. Findings from studies suggest increased activity in right-sided temporal regions after LSVT-LOUD® in participants with Parkinson’s disease.^15-18^ However, the studies were heterogeneous in terms of neuroimaging modality and all included a rather small number of participants. Consequently, additional research is necessary to further validate these findings.

Resting-state functional MRI (rsfMRI) can be used to identify the intrinsic network architecture and is not biased by task performance. In Parkinson’s disease, structural brain changes occur later in the disease being preceded by changes in connectivity and metabolism.^19^ Thus, rsfMRI might be a better neuroimaging marker for Parkinson’s disease. In neurodegenerative diseases, spectral dynamic causal modelling is suitable for assessing effective brain network connectivity.^20,21^ This type of analysis enables estimation of the direct, causal effect of one neuronal population on another. Thus, it is possible to investigate alterations in a pre-specified network and to quantify changes in brain activity in a pre- to post-interventional design. These facts make dynamic causal modelling suitable for our study design.

The aim of this study was to evaluate the effects of HiCommunication on a broad spectrum of acoustic measures of speech and voice as well as on effective connectivity using rsfMRI. Spectral dynamic causal modelling and the parametric empirical Bayes method were used to describe the effective connectivity of a speech-motor-related network in people with mild-moderate Parkinson’s disease.^15^ We further related the statistically significant speech changes to changes in effective brain connectivity following the intervention.

## Methods

### Participants

Ninety-five people with mild-moderate Parkinson’s disease from the RCT EXPANd were included; 47 who participated in the speech and voice intervention, HiCommunication, and 48 who participated in an active control intervention (Table 1).^13,14^ For power calculations, randomization, allocation methods and blinding procedures, see the study protocol.^13^ The inclusion criteria were: diagnosed with idiopathic Parkinson’s disease, Hoehn and Yahr 2–3, age ≥ 60 years, a Montreal Cognitive Assessment (MoCA)^22^ score ≥ 21 and ≤27 on the Mini Balance Evaluation Systems Test (MiniBESTest).^23^ The RCT was performed in line with the principles of the Declaration of Helsinki, approved by the Regional Ethical Review Board Stockholm (2016/1264-31/4, 2017/1258-32, 2017/2445-32) and performed in a university, a university hospital and follow-up partially in the participants’ homes due to COVID-19. The participants received written and oral information and provided written informed consent. All participants were assessed in their ON stage and asked to not alter medication during the study period. The levodopa-equivalent daily dosage (LEDD) was calculated.

**Table 1:** Descriptive data (observed values) of the intention-to-treat cohort (*n* = 95)

Descriptive	HiCommunication *n* = 47	HiBalance *n* = 48
Age (years)^a^	71.1	71.0
	(6.4)	(5.9)
Sex		
Female^b^	17 (36.2%)	18 (37.5%)
Male^b^	30 (63.8%)	30 (62.5%)
Hoehn and Yahr^c^	2	2
	(1)	(0)
MDS-UPDRS III^a^	31.4	31.2
	(10.1)	(11.9)
MDS-UPDRS^a^	50.2	51.0
	(15.6)	(18.8)
Years since PD diagnosis^a^	3.7	4.8
	(4.3)	(4.4)
PDQ 39^a^	18.6	22.6
	(11.7)	(12.3)
MoCA^a^	25.5	26.1
	(2.5)	(2.3)
LEDD^a^	430.0	551.0
	(286.2)	(604.8)
Dysarthria test^a^	0.2	0.2
	(0.2)	(0.2)
Presence intervention (%)^c^	85%	85%
	(22.5%)	(25.0%)

SD, standard deviation; IQR, interquartile range; PD, Parkinson's disease; MDS-UPDRS, Movement Disorders Society—Unified Parkinson's Disease Rating Scale; PDQ-39, the Parkinson's Disease Questionnaire-39; higher scores reflect a higher Parkinson's disease-specific health-related quality; MoCA, Montreal Cognitive Assessment, higher scores reflect a higher level of global cognitive function; LEDD, levodopa-equivalent daily dosage. The Dysarthria test is rated on a scale from 0 = no speech deviation to 3 = severe speech deviation, higher scores represent a more severe degree of dysarthria.^24^ Presence intervention (%): attendance in the group intervention sessions. ^a^Mean (SD). ^b^n (%). ^c^Median (IQR).

### Adherence and missing data

**Figure 1:**
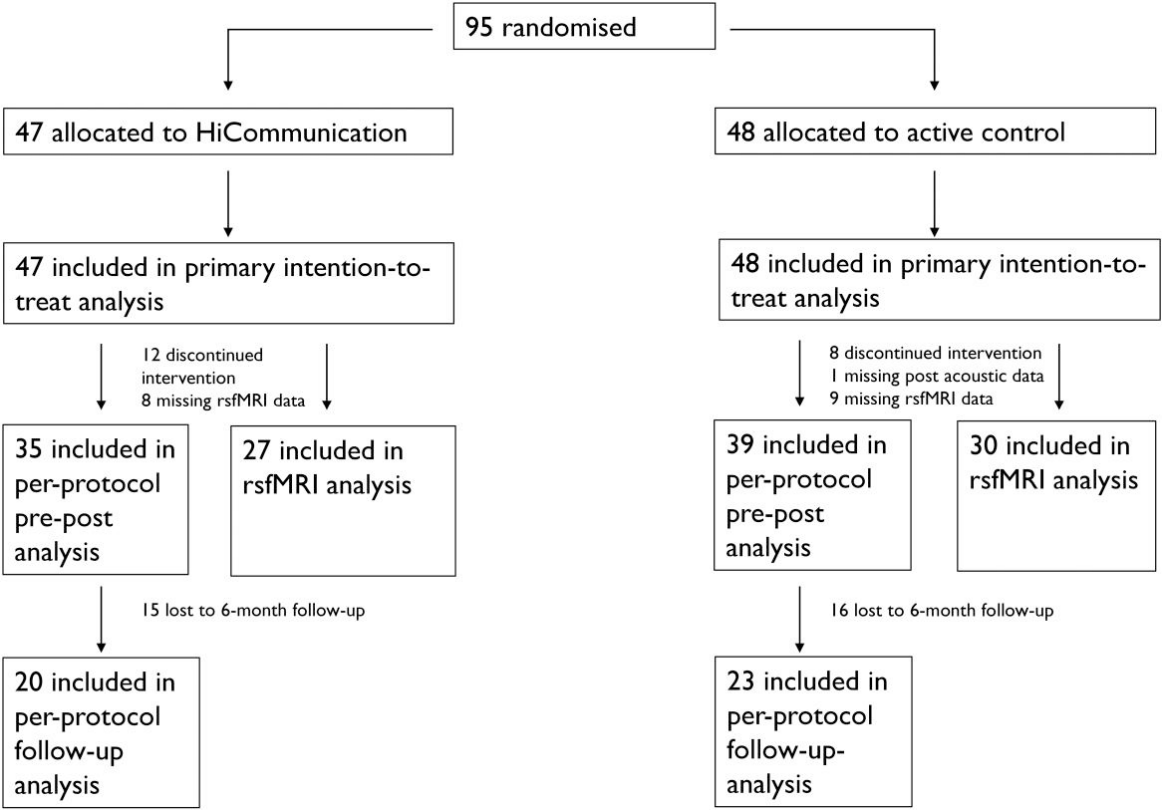
**Trial flowchart.** Details of study flow. rsfMRI, resting-state functional magnetic resonance imaging.

Twenty participants within the RCT discontinued participation, resulting in a total attrition rate of 21%: 26% for the HiCommunication group and 17% for the active control group. No adverse events were reported during the HiCommunication intervention, and eight adverse events were reported during the active control intervention for balance and gait.^14^ Fig. 1 shows the trial flow chart and number of participants included in each analysis. For detailed information on missing data, see Supplement.

### Intervention

HiCommunication is an intervention based on principles that are thought to promote experience-dependent neural plasticity such as intensity, repetition, specificity and saliency.^25^ The format is a 10-week group intervention consisting of three sessions per week, each lasting 1 hour. Two sessions are conducted in the clinic with a speech-language pathologist in groups of six to eight participants, and one session involves home training supported by a training diary. The core areas of HiCommunication are voice intensity and articulatory precision, with the objective to practice loud and clear speech. Two other core areas are word retrieval and memory, primarily incorporated to introduce cognitively more complex content into the intervention. The intervention is built in a hierarchical structure with a gradual progression of cognitive loading (Supplementary Table 1).^12,13^ Participants randomized to the active control intervention (HiBalance) participated in balance and gait group training with the same format and hierarchical structure.^26^ The core areas of HiBalance are sensory integration, motor agility, anticipatory postural adjustments and stability limits.

### Acoustic analysis

#### Speech recordings

Speech recordings were performed pre- and post-intervention according to standardized routines for high-quality recordings in a sound-proof recording studio with the equipment Sony Digital Audio Tape Deck DTC-ZE700. Due to the COVID-19 pandemic, for a subgroup of the participants, six-month follow-up speech recordings were performed in the participants’ homes. A portable recording system (Focusrite 2i2 and a sound level calibrator, Focusrite USB driver 4.63.24.564) was used for the home recordings. Sopran (version 1.0.22© Tolvan Data), a software for sound processing and analyses, and a head microphone (Sennheiser HSP 4 with an MZA 900 P phantom power adapter) that was calibrated for a pre-determined mouth-microphone distance of 15 cm were used for all recordings. The recording protocol included the speech tasks maximum sustained phonation, rapid syllable repetition, reading of sentences, a Swedish standardized phonetically balanced text (289 syllables) constructed for evaluation of neuromotor speech disorders as well as elicitation of speech monologue.^3,27^

#### Outcome variables and analysis procedure

The primary outcome measure was voice sound level in text reading (Table 2). Additional acoustic measures were chosen based on: (i) a spectrum of speech dimensions targeted by the intervention grouped into the broader speech domains voice function, voice quality, articulation and prosody; (ii) the presence of speech and voice symptoms within our Parkinson's disease cohort identified by perceptual analysis during a session with consensus discussions by three speech-language pathologists with substantial experience in motor speech disorders (co-authors H.S., E.S. and J.K.G.); (iii) a recent review by Rusz *et al*.^24^ in which acoustic methods of analyses with the ability to differentiate hypokinetic or hyperkinetic dysarthria from healthy speech and voice production are presented and discussed.

**Table 2:** Acoustic outcomes of HiCommunication sorted under respective speech domain and dimension

Speech domain	Speech dimension	Acoustic outcome (unit)	Speech task	Analysis software
Voice function	Mean vocal loudness in text reading	Voice sound level in text reading (dBC)^a^	Text reading	Sopran (version 1.0.22 © Tolvan Data)
	Mean vocal loudness in monologue	Voice sound level in monologue (dBC)	Speech monologue elicited by the written instruction: ‘speak about something you like or like to do’.	Sopran (version 1.0.22 © Tolvan Data)
	Mean vocal loudness in text reading in noise	Voice sound level in noise (dBC)	Reading of the initial 142 syllables of the standardized text whilst pink noise (70–72 dB) was played in headphones (Sony MDRZX660AP)	Sopran (version 1.0.22 © Tolvan Data)
	Speech phonation	Maximum phonation time (MPT) (s)	Sustained vowel [a:]	Sopran (version 1.0.22 © Tolvan Data)
Voice quality	General dysphonia	Acoustic Voice Quality Index (AVQI)	Text reading and sustained vowel [a:]	Analysis tool AVQI (version 01.03, Phonanium, 2021)
	Proportion of harmonic sound to noise in the voice	Harmonics-to-Noise Ratio (HNR) (dB)	Text reading and sustained vowel [a:]	Analysis tool AVQI (version 01.03, Phonanium
Articulation	Bilabial articulatory diadochokinesis (DDK), alternating motion rates (AMR)	DDK-AMR (syllables/s)	Repetition of the syllable sequence /pa-pa-pa/	Sopran (version 1.0.22 © Tolvan Data)
	Articulatory diadochokinesis (DDK), sequential motion rates (SMR)	DDK-SMR (syllables/s)	Repetition of the syllable sequence /pa-ta-ka/	Sopran (version 1.0.22 © Tolvan Data)
	Vowel articulation	Vowel articulation Index (VAI)	Sentences and text reading	Praat (version 6.0.36)
Prosody	Standard deviation (SD) of Fundamental frequency (F0) contour	F0 SD (semitones)	Text reading	Praat (version 6.0.36)^28^

dB, decibel; dBC, C-weighted decibel; s, seconds. ^a^Main outcome measure.

Extractions from the speech recordings of each participant at each time point (pre, post, follow-up) were used for acoustic analysis. Table 2 includes an overview of analysis procedures for each acoustic outcome, respectively. The analysis procedure was performed blinded to time and intervention condition. See Supplement for detailed information on acoustic outcome variables and analysis procedures.

#### Reliability

The intra-class correlation coefficient (ICC) was computed to assess the consistency between two separate acoustic analyses performed by the same assessor in a randomly selected subgroup (*n* = 20) out of all participants. The ICC was calculated for all acoustic variables except for the Harmonics-to-Noise Ratio (HNR). However, since the acoustic analysis for HNR was performed within the Acoustic Voice Quality Index (AVQI) analysis, the ICC can be expected to be similar for both measures. For one participant the voice sound level in noise was missing, hence the ICC was computed using data from 19 participants. There was an excellent consistency between the ratings for all variables, using the two-way random effects model and ‘single rater’, supporting the stability of the measures (Supplementary Table 2).^29^

#### Statistical analyses

All acoustic data were inspected prior to statistical analyses. Since the fundamental frequency (F0) variability was right skewed, it was log-transformed towards normality. The first-level analyses of the acoustic outcomes included data from all participants (intention-to-treat) with missing values imputed using multiple imputation. The R package ‘mice’ and the predictive mean matching method were used with 30 imputed data sets, 20 iterations and data separately imputed for the two groups (R version 4.2.3, R studio version 2023.03.0, mice version 3.15.0^30^). Predictors for each outcome were chosen based on theoretical assumptions in combination with correlation coefficients (Supplementary Table 3). The imputations were evaluated using diagnostic plots.

Linear multilevel models were used for both intention-to-treat (*n* = 95) and complementary per-protocol analyses, where only participants who attended at least 60% of the intervention sessions were included (*n* = 74). Group and time (pre = 0, post = 1) and their interaction were used as predictors. In addition, per-protocol six-month follow-up analyses (*n* = 43) were performed where group and time (pre = 0, post = 1, follow-up = 2) and their interaction were used as predictors. The alpha level for all models was set to 0.05, two-sided. The models were specified with the pre- and post-values as level 1, clustered within the individuals, i.e. level 2, with group as a factor on level 2. We allowed for random intercepts but not random slopes. Time and group and their interaction were used as independent variables. We compared the intention-to-treat models with and without the covariates sex and presence in the intervention (in %) using the Akaike Information Criteria (AIC). Since the AIC did not improve evidently and the results [rounded numbers of estimates, *P*-values and confidence intervals (CIs)] were not altered when adding the covariates, the simple models without covariates were chosen for all acoustic outcomes.

We employed restricted maximum likelihood estimation for our linear multilevel models. Given the lack of consensus on the accurate calculation of degrees of freedom for multilevel models and the susceptibility of suggested methods to errors, we opted not to report degrees of freedom. In our intention-to-treat analyses, we utilized the R package ‘mice’ to estimate multilevel models for each imputed data set, followed by pooling the estimates. Since back-transformation of estimates of log-transformed models can introduce bias, and there were no considerable group-by-time effects in log-transformed F0 variability, we refrained from back-transforming the estimates.

Since effect sizes for multilevel regressions should be interpreted with caution due to the complexity of the models, we chose to solely report estimated coefficients for each predictor along with CIs that provides information about the strength and direction of association between predictors and the outcome variable.^31,32^

A clinically relevant change in voice sound level in text reading was defined as a change of ≥2 C-weighted decibel (dBC) based on a study by Fox and Ramig^33^ where participants with Parkinson’s disease used a voice sound level that was 2–4 decibel (dB) lower compared to healthy controls. Clopper–Pearson’s exact 95% CIs for the proportion of participants with a clinically relevant increase in voice sound level in text reading were calculated in Microsoft Excel (2016), as well as for the proportion of participants with a reliable change (e.g. not due to measurement error) (a decrease of ≥0.54) in AVQI.^34^

Throughout the acoustic analysis of the results from this study, we chose not to correct for multiple comparisons to reduce the risk of making Type II errors, given that all comparisons were hypothesis-driven and pre-determined. Instead, we report all individual *P*-values and CIs to enable informal consideration of multiple comparisons when interpreting the results.^35,36^

### Clinical and disease-related measurements

To capture Parkinson’s disease severity, the Movement Disorder Society Unified Parkinson’s Disease Rating Scale (MDS-UPDRS) was assessed pre- and post-intervention.^37^ As a measure of global cognitive function, the MoCA was applied and used as an inclusion criterion in the RCT.^22^ Self-reported data on health-related quality of life were collected using Parkinson’s Disease Questionnaire-39 (PDQ-39).^38^ Dysarthria was assessed using The Dysarthria Assessment, a Swedish standardized clinical test.^27^

### MRI analyses

#### Acquisition and pre-processing

The rsfMRI was acquired with an echo-planar imaging (EPI) sequence with repetition/echo time (TR/TE) = 2073/7.3 ms, 224 volumes, 40 slices, 75° flip angle, a voxel-size of 3.5 mm^3^ and eyes open looking at a fixation cube. The acquisition took place 1–3 weeks pre- and post-intervention. We analysed 27 HiCommunication participants and 32 active controls pre- and post-intervention. The images were pre-processed through the HiveDB^39^ using the SPM12 standard pipeline for rsfMRI (MATLAB R2019b, SPM12 version 7771) i.e. images were reoriented, slice time corrected, realigned, co-registered with their T1 images, normalized and smoothed (full width at half-maximum, FWHM = 8 mm^3^). Each participant’s co-registered white matter and cerebrospinal fluid segmented images were binarized with a threshold of 0.3 in FSL (version 6.0.3) and used as a mask to obtain nuisance regressors. To obtain five white matter and five cerebrospinal fluid regressors, we applied the aCompCor approach using the function of Mascali *et al*.^40^ The Friston 24-model^41^ was chosen for motion regression: six rigid body motion parameters, their squares, their temporal derivatives and the squares of the temporal derivatives.^42^ A linear model was constructed to regress out nuisance.^40^

#### Regions of interest

We used regions of interest (ROIs) to study a speech-motor-related network defined according to a study by Baumann *et al*.^15^ This study investigated the effects of LSVT-LOUD®, an intervention comparable to HiCommunication, on brain functional connectivity in people with Parkinson’s disease (*n* = 11). Brain activity was assessed pre- and post-LSVT-LOUD® during a reading task in the MRI scanner and compared between people with Parkinson’s disease and healthy controls. Hypoactivity was found in the following ROIs pre-intervention: left and right supplementary motor areas (MNI: *x* = 12, *y* = −8, *z* = 64) (*x* = −10, *y* = 0, *z* = 68) and left paracentral gyrus (*x* = −2, *y* = −14, *z* = 68). To extract the signal of the ROIs, we built a general linear model with smoothed, and nuisance-regressed rsfMRI files using age and sex as covariates. A threshold of *P* < 0.05 (uncorrected) was set to remove any spurious activity. This procedure is at the first level, performed for each subject. ROI time series were extracted as the first principal eigenvariate of all voxels in a 6 mm sphere.

#### Effective connectivity analysis

To assess brain network effective connectivity, spectral dynamic causal modelling can be applied.^43-45^ It enables estimation of the directed, causal effect of one neuronal population on another. The resulting effective connectivity between ROIs is directed and measured in Hertz (Hz). Positive values indicate excitation, while negative values indicate inhibitory influences. A Bayesian framework—parametric empirical Bayes—is then used to infer a neuronal interaction model, at the group level, that fits the subject-level connectivity parameters the best.^46^ We performed first-level analyses to estimate the connectivity strength within each participant and time point by a fully connected model, i.e. using all possible connections among the ROIs. For spectral dynamic causal modelling, each participant's model was estimated to provide a probability density over the connection strengths and a lower bound approximation of the (log) model evidence (known as free energy) to evaluate the quality of the model fitting. The participants’ effective connectivity parameters were then taken to the second-level analysis (group level), where all parameters are modelled by a (Bayesian) general linear model.

For the second-level analysis, we built a hierarchical model as the primary model, to analyse the main effect of time (pre- and post-intervention), the main effect of group (HiCommunication and active controls) as well as the group-by-time interaction. In case of a significant group-by-time interaction effect, in *post hoc* analyses, we also built a second-level model on the pre- and post-HiCommunication intervention. This estimated the group, i.e. pre- and post-intervention, mean of the parameters. Using parametric empirical Bayes, the hypothesis was tested by comparison of the fully connected model to the reduced models. Connection strengths and connection changes with a Bayesian posterior probability Pp > 0.95 were regarded as statistically robust effects. The second-level models included LEDD, and the hierarchical model also included age, as regressors of no interest.

For regional clinico-functional relations, we calculated associations between effective connectivity parameters and behavioural measures where a change pre to post-intervention was found (defined by a difference in mean at a 95% CI) using parametric empirical Bayes routines. We extracted the effective connectivity parameters of the winning model for association analysis in which the effective connection strength was associated with the acoustic measures of speech and voice.

### Deviations from preregistration

Our analysis plan was preregistered on OSF (https://osf.io/s952g/) and deviations are listed in the Supplement.

## Results

### Acoustic outcomes of HiCommunication

#### Primary intention-to-treat analysis

**Figure 2:**
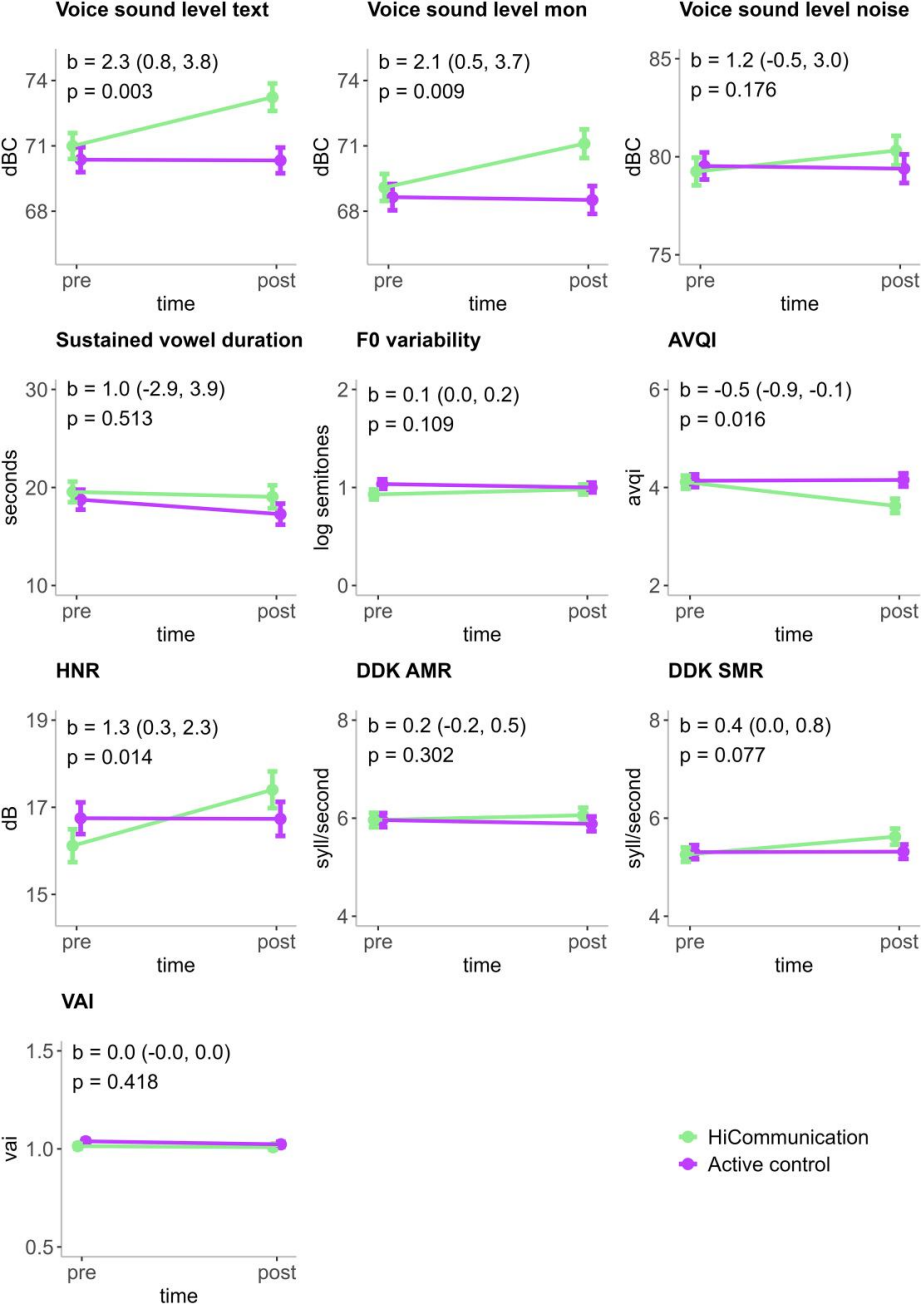
**Acoustic outcomes of HiCommunication.** The mean values, the standard error of the mean (error bars) as well as the *b*-values, e.g. the unstandardized estimates of the time by group interaction, and their 95% confidence intervals (CIs), are predicted values based on the intention-to-treat analyses (imputed data in linear mixed-effects models, *n* = 95). Voice sound level mon: voice sound level in monologue. F0 variability: fundamental frequency standard deviation in semitones (log-transformed), higher values indicate a more varied pitch. AVQI: Acoustic Voice Quality Index, scores above 2.95 indicate dysphonia; HNR: Harmonics-to-Noise Ratio, higher values indicate stronger tonal components compared to noise in the signal, DDK-AMR and DDK-SMR: diadochokinetic rate alternating and sequential motion rates in syllables per second (syll/second), VAI: Vowel Articulation Index; higher values indicate better articulatory ability and clarity in vowel production. dBC, C-weighted decibel; log, logarithmic scale; pre, pre-intervention; post, post-intervention.

There was a statistically significant group-by-time interaction effect for our primary outcome, voice sound level in text reading (unstandardized regression coefficient (*b*) = 2.3 [95% CI = 0.8, 3.8], *P* = 0.003) (Fig. 2 and Table 3). Thus, we found a higher voice sound level in text reading post-HiCommunication compared to post the active control intervention. Further, there were statistically significant group-by-time interaction effects in favour of HiCommunication for our secondary acoustic outcomes voice sound level in monologue (*b* = 2.1 [95% CI = 0.5, 3.7], *P* = 0.009), AVQI (*b* = −0.5 [95% CI = −0.9, −0.1], *P* = 0.016) and HNR (*b* = 1.3 [95% CI = 0.3, 2.3], *P* = 0.014). There were no statistically significant differences for any of the other acoustic outcomes.

**Table 3:** Descriptive data (observed values) and analyses estimates (by intention-to-treat) for the acoustic outcomes

	HiCommunication		Active control			Group		Time		Group:time interaction		Intercept	
	Pre	Post	Pre	Post	Reg.	*b* (95% CI)	*P*	*b* (95% CI)	*P*	*b* (95% CI)	*P*	*b* (95% CI)	*P*
Voice sound level text	70.8	73.0	70.3	70.6	lin.	0.6	0.449	0	0.944	2.3	**0.003**	70.4	<0.0001
	(4.0)	(4.0)	(3.5)	(3.9)		(−1, 2.3)		(−1, 0.9)		(0.8, 3.8)		(69.2, 71.5)	
Voice sound level monologue	69.0	71.2	68.6	68.8	lin.	0.4	0.605	−0.1	0.814	2.1	**0.009**	68.6	<0.0001
	(3.7)	(4.3)	(4.2)	(4.3)		(−1.3, 2.2)		(−1.2, 1)		(0.5, 3.7)		(67.5, 69.8)	
Voice sound level noise	79.2	80.5	79.5	79.9	lin.	−0.3	0.776	−0.1	0.83	1.2	0.176	79.5	<0.0001
	(4.3)	(3.8)	(5.2)	(5.2)		(−2.2, 1.7)		(−1.4, 1.1)		(−0.5, 3)		(78.2, 80.9)	
Sustained vowel duration	19.5	19.2	18.8	17.2	lin	0.8	0.587	−1.5	0.146	1	0.513	18.8	<0.0001
	(7.1)	(7.4)	(6.7)	(7.2)		(−2.1, 3.7)		(−3.5, 0.5)		(−2, 3.9)		(16.7, 20.8)	
F0 variability	2.4	2.4	2.6	2.7	log lin.	−0.1	0.13	0	0.346	0.1	0.109	1	<0.0001
	(0.7)	(0.9)	(0.9)	(0.7)		(−0.2, 0)		(−0.1, 0)		(0, 0.2)		(0.9, 1.1)	
AVQI	4.1	3.6	4.1	4.1	lin.	0	0.88	0	0.909	−0.5	**0.016**	4.1	<0.0001
	(1.1)	(1.0)	(0.7)	(0.7)		(−0.4, 0.3)		(−0.3, 0.3)		(−0.9, −0.1)		(3.9, 4.4)	
HNR	16.1	17.6	16.7	17.0	lin.	−0.6	0.231	0	0.966	1.3	**0.014**	16.7	<0.0001
	(2.7)	(2.8)	(2.4)	(2.0)		(−1.7, 0.4)		(−0.7, 0.7)		(0.3, 2.3)		(16, 17.5)	
DDK-AMR	6.0	6.2	6.0	6.0	lin.	0	0.994	−0.1	0.518	0.2	0.302	6	<0.0001
	(0.9)	(0.8)	(1.1)	(0.9)		(−0.4, 0.4)		(−0.3, 0.2)		(−0.2, 0.5)		(5.7, 6.2)	
DDK-SMR	5.3	5.6	5.3	5.3	lin.	0	0.807	0	0.943	0.4	0.077	5.3	<0.0001
	(0.9)	(1.1)	(0.9)	(1.1)		(−0.5, 0.4)		(−0.2, 0.3)		(0, 0.8)		(5, 5.6)	
VAI	1	1	1	1	lin.	0	0.157	0	0.075	0	0.418	1	<0.0001
	(0.1)	(0.1)	(0.1)	(0.1)				(−0.1, 0)		(0, 0)		(1, 1.1)	

The pre- and post-values are mean and standard deviation for all normally distributed outcomes and otherwise median and interquartile range (F0 variability). The column Reg. defines the type of multilevel model (mlm) used for the outcome; lin., linear; log lin., linear mlm on logged values; *b*, unstandardized estimate; CI, confidence interval; in bold: *P* <0.05. F0 variability: fundamental frequency standard deviation in semitones (log-transformed), higher values indicate a more varied pitch. AVQI: Acoustic Voice Quality Index, scores above 2.95 indicate dysphonia, HNR: Harmonics-to-Noise Ratio, higher values indicate stronger tonal components compared to noise in the signal, DDK-AMR and DDK-SMR: diadochokinetic rate alternating and sequential motion rates in syllables per second, VAI: Vowel Articulation Index; higher values indicate better articulatory ability and clarity in vowel production.

#### Per-protocol analysis

Per-protocol analyses were in line with the intention-to-treat analyses (Supplementary Table 4). In addition, there was a statistically significant group effect for log-transformed F0 variability (*b* = −0.2 [95% CI = −0.3, 0.0], *P* = 0.047).

#### Clinically relevant change in voice sound level

**Figure 3:**
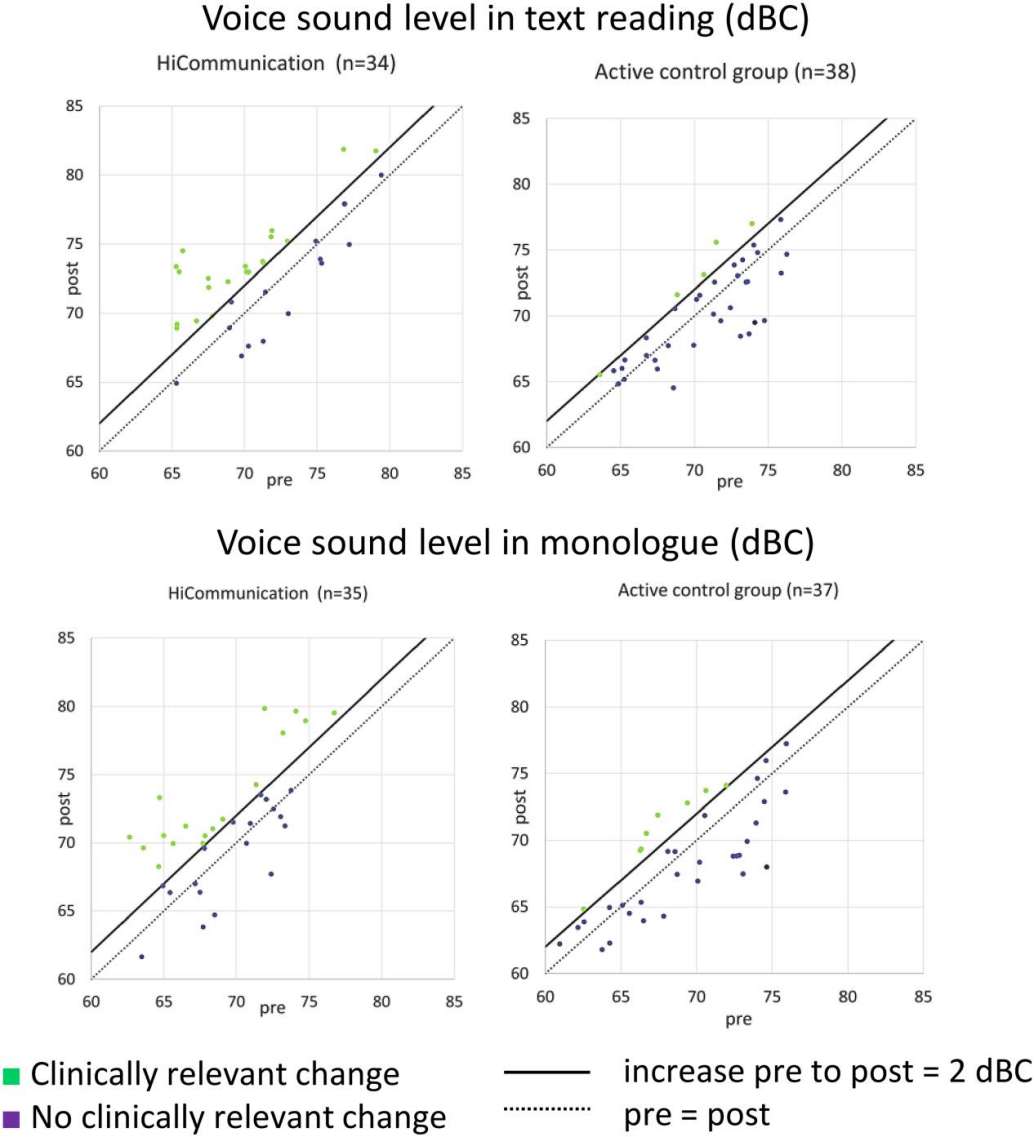
**Brinley plots of the change in voice sound level in text reading and in monologue (dBC) after intervention with regard to clinical relevance (increase pre- to post-intervention ≥ 2 dBC) (*n* = 72).** The participants’ point estimates, and their distributions are observed values. Each participant with both a pre- and post-value is plotted in the graph. dBC, C-weighted decibel; pre, pre-intervention; post = post-intervention.

As complementary analyses, we calculated Clopper–Pearson exact CIs for the proportion of participants with a clinically relevant change in voice sound level (≥2 dBC). The percentage of participants with a clinically relevant increase in voice sound level in text reading post-intervention was 59%, CI [41%, 75%] for HiCommunication and 13%, CI [4%, 28%] for active controls. The clinically relevant increase in voice sound level in monologue post-intervention was 49%, CI [31%, 66%] for HiCommunication and 22%, CI [10%, 38%] for active controls (Fig. 3).

#### Reliable change in Acoustic Voice Quality Index

**Figure 4:**
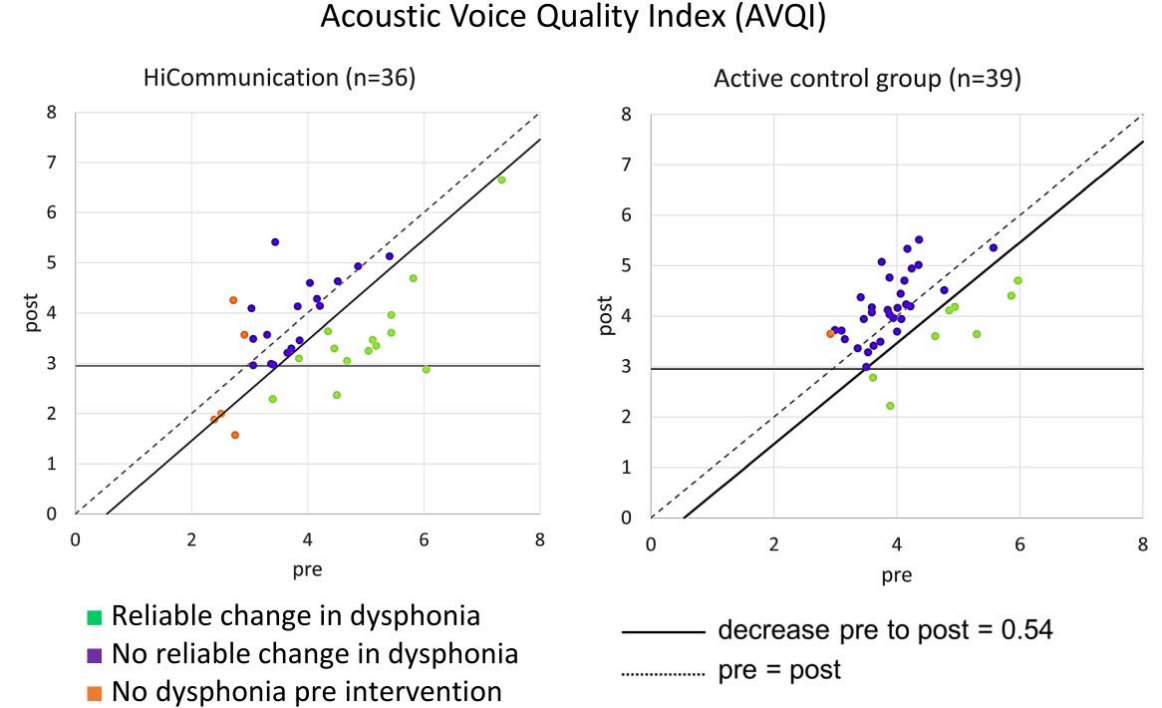
**Brinley plots of the change in AVQI score after intervention with regard to reliability (decrease pre- to post-intervention ≥ 0.54) (*n* = 75).** The participants’ point estimates, and their distributions are observed values. Each participant with both a pre- and post-value is plotted in the graph. AVQI, Acoustic Voice Quality Index; pre, pre-intervention; post, post-intervention.

Pre-intervention, the mean score of AVQI was 4.1 (SD 1.1) for the HiCommunication group and 4.1 (SD 0.7) for the active control group, thus exceeding the cut-off for dysphonia (2.95) in both groups (Table 3). Since the estimate of the mean decrease (see intention-to-treat analysis above) was lower than the limit for what is considered a reliable change (e.g. not due to measurement error), we also calculated Clopper–Pearson exact CIs for the proportion of participants with a reliable change in AVQI. The proportion of participants with a reliable decrease in AVQI was 39%, CI [23%, 57%] after intervention with HiCommunication and 21%, CI [9%, 36%] after the active control intervention (Fig. 4).

#### Six-month follow-up

We performed per-protocol follow-up analyses to investigate whether the positive changes in the acoustic measures remained six months post-intervention. Also in this cohort (*n* = 43), there were statistically significant group-by-time interaction effects for voice sound level in text reading (*b* = 1.8 [95% CI = 0.2, 3.5], *P* = 0.034), voice sound level in monologue (*b* = 1.8 [95% CI = 0.1, 3.6], *P* = 0.048), AVQI (*b* = −0.5 [95% CI = −0.9, −0.5], *P* = 0.025) and HNR (*b* = 1.4 [95% CI = 0.4, 2.5], *P* = 0.010) pre- to immediately post-intervention. Pre-intervention to follow-up after six months, there was a statistically significant group-by-time interaction effect for log-transformed F0 variability (*b* = 0.2 [95% CI = 0.0, 0.3], *P* = 0.045). There were no statistically significant group-by-time interaction effects between either time points for the remaining acoustic measures (Supplementary Table 5).

### Effective connectivity

**Figure 5:**
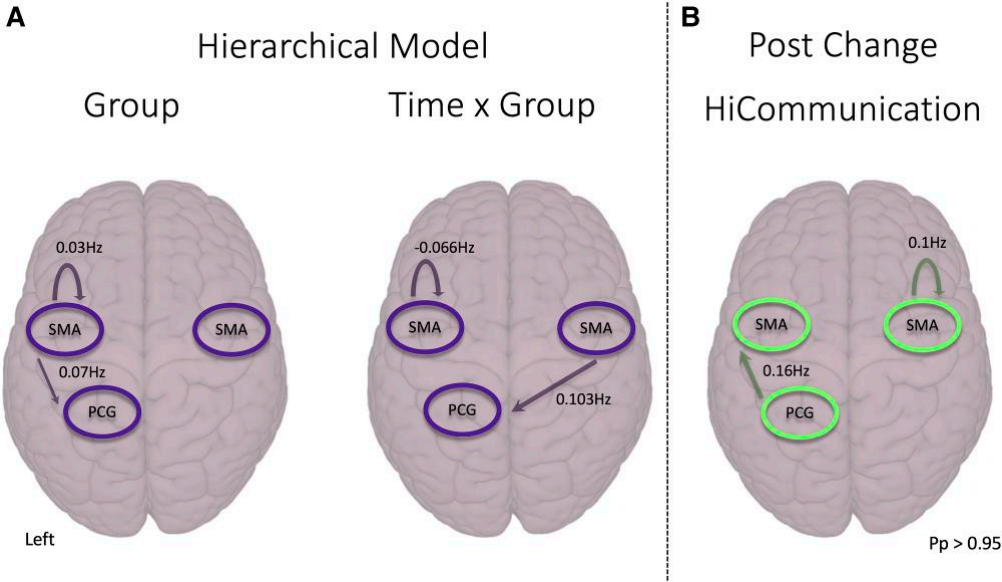
**Second-level parametric empirical Bayes routines model of effective brain connectivity changes in people with Parkinson’s disease who participated in HiCommunication (*n* = 57).** (**A**) Hierarchical model to compare HiCommunication intervention versus active controls. The effects of group-by-time interaction, group and time were modelled and only the statistically significant connections are shown. (**B**) *Post hoc* analyses comparing changes pre- versus post-HiCommunication intervention. Analyses were corrected for levodopa-equivalent daily dosage and the hierarchical model additionally for age. Images are shown in neurological convention, the left is on the left side. Only results above Pp > 0.95 are shown here. SMA, supplementary motor area; PCG, paracentral gyrus; Pp, posterior probability.

Statistically significant group-by-time interaction effects showed decreased inhibitory self-connectivity of the left supplementary motor area (−0.066 Hz) i.e. disinhibition, and increased effective connectivity from the right supplementary motor area to the left paracentral gyrus (0.103 Hz) post-HiCommunication (Fig. 5). The main effect of the group showed a significantly increased inhibitory self-connectivity of the left supplementary motor area (0.03 Hz) and increased connectivity from the left to the right supplementary motor area (0.07 Hz) in HiCommunication compared to the active controls. There was no significant effect of time.

In *post hoc* parametric empirical Bayes analyses, we analysed the change in connectivity from pre- to post-HiCommunication. Post-HiCommunication, excitatory effective self-connectivity in the right supplementary motor area (0.1 Hz) and increased connectivity between the left paracentral gyrus and the left supplementary motor area (0.16 Hz) were found.

Note that we here only report the connections that are above the statistical threshold of posterior probability Pp > 0.95 (considered a strong evidence).

### Associations between effective connectivity and behavioural change

We used parametric empirical Bayes to relate a group level effective connectivity change to speech and voice variables with a statistically significant difference pre- to post-HiCommunication, i.e. voice sound level in text reading and monologue, AVQI and HNR. None of the associations were statistically significant.

## Discussion

### Main findings

We showed positive effects on voice sound level and measures of voice quality (AVQI and HNR) following a novel intervention for speech and communication—HiCommunication. Notably, the effect on the primary outcome measure voice sound level in text reading was clinically relevant. The effects were not retained at the follow-up after six months. Furthermore, we showed both increases and decreases in effective connectivity in the supplementary motor area and paracentral gyrus post-HiCommunication. However, we could not identify any associations between speech and voice changes and brain effective connectivity.

### Intervention effects on voice function

Voice intensity is one of the core areas of HiCommunication where the use of a loud voice with good voice quality is practiced with the goal to maintain an adequate voice sound level in communicative situations.^12^ Consequently, improvements in voice sound level and voice quality were hypothesized. The mean changes were somewhat smaller compared to effects shown after LSVT-LOUD®.^8,11^ This could be due to several causes. First, the level of intensity differs between HiCommunication and LSVT-LOUD®, with a total of 20 hours as well as 10 hours of home training over 10 weeks, compared to 16 hours over 4 weeks and daily home training. HiCommunication was developed based on the clinical experience that many individuals with Parkinson’s disease find it challenging to travel to the clinic four times per week, and as a compromise to limit healthcare resources.^12^ Second, in contrast to LSVT-LOUD® that primarily focuses on vocal loudness, HiCommunication focuses on voice function (loudness and quality) and articulatory precision (clear speech), Third, HiCommunication is delivered in a group to meet the desire of people with Parkinson’s disease to have a stronger focus on psychosocial interaction.^12,47^ Although this could potentially lead to a reduced exercise dose per intervention session and individual, the communicative situations intrinsic in the group setting are hypothesized to lead to a higher degree of retention and transfer of treatment effects to communicative situations outside the clinical setting. Fourth, in this study, the inclusion criterium regarding speech and voice was solely to have at least one self-reported speech and voice symptom. Therefore, our cohort presented with generally mild dysarthria that could be a reason for smaller effects post-HiCommunication at a group level. Of note, participants with a lower voice sound level pre-HiCommunication generally showed larger increases in voice sound level post-HiCommunication, and thus seemed to benefit the most from the intervention. Furthermore, participating in intervention when speech and voice symptoms are still rather mild may be beneficial due to the progressive nature of Parkinson’s disease symptoms and associated cognitive difficulties. This indicates the ecological validity of the study.

The mean decrease in AVQI post-HiCommunication was −0.5 (group-by-time effect). Since the threshold for a reliable change (i.e. not due to measurement error) is −0.54, we also conducted complementary analysis and found a trend of a reliable decrease in AVQI post-HiCommunication. A decrease in AVQI was shown also in a study evaluating the effects of LSVT-LOUD®.^48^ Notably, our participant cohort exceeded the cut-off for dysphonia pre-intervention that was not the case in that study. Considering the sparse number of studies using AVQI as an outcome measure of improvements in voice quality following speech intervention in Parkinson’s disease, additional studies are needed to evaluate the validity.

Harmonics-to-Noise Ratio may differ between people with Parkinson’s disease and healthy controls.^24^ It has been sparsely used as an outcome measure of Parkinson’s disease speech intervention but has been shown to be improved following deep-brain stimulation in people with Parkinson’s disease.^49^ We found an improvement in Harmonics-to-Noise Ratio post-HiCommunication. However, the clinical relevance of the magnitude of change still needs to be investigated.

#### Clinical relevance of changes in voice sound level

Importantly, we found that the positive change in vocal loudness post-HiCommunication was clinically relevant for many of the participants (59%). Effects on vocal loudness, generally the primary outcome measure of speech and voice intervention in Parkinson’s disease, have typically been defined based on statistical comparisons of mean changes in voice sound level pre- to post-intervention.^7-9^ This is problematic since such statistical tests provide no information on the individual variability of the treatment response and because statistical significance does not imply clinical significance.^50^ To the best of our knowledge, no attempts have been made to establish the minimal clinically important difference (MCID), i.e. the smallest change in vocal loudness post-intervention that an individual would deem important.^51,52^ Here, we presented the effect on voice sound level in text reading and monologue in terms of clinical relevance using a threshold based on a study showing that participants with Parkinson’s disease use a voice sound level that is 2–4 dB lower compared to healthy controls.^33^ However, we encourage more research in this area targeted to establish the MCID in vocal loudness, and other acoustic outcomes of speech and voice, using anchor-based methods to bring the person with Parkinson’s disease's perspective to prominence. Of importance is that additional studies in this project are evaluating speech and voice changes with audio-perceptual methods and self-reported data on speech, voice and communication. This strengthens the potential of evaluating outcomes of HiCommunication with regard to clinical relevance.

### Additional acoustic outcomes

We found no statistically significant changes in the acoustic outcomes within the speech domains articulation and prosody, respectively, or in two out of four acoustic outcomes within the speech domain voice function: voice sound level in noise and maximum phonation time. Early in the disease progression changes in voice function may be the most prominent symptoms of hypokinetic dysarthria.^1,53,54^ Considering the generally mild dysarthria in our cohort, effects primarily on voice function and -quality post-HiCommunication were somewhat expected. Furthermore, the absence of significant changes in acoustic outcomes concerning articulation and prosody might stem from the presence of diverse speech phenotypes, each exhibiting varied responses to levodopa.^55^ Prosodic features such as F0 variability could be well corrected by levodopa. Further studies in cohorts representing a population of more moderate dysarthria are motivated to evaluate the post-intervention effects on speech and voice symptoms relating to all speech domains affected in hypokinetic dysarthria.

#### Intervention effects on articulation

There were no effects in the acoustic measure of vowel articulation (Vowel Articulation Index) even though articulatory precision is one of the target areas of HiCommunication.^12^ However, changes in articulation observed in Parkinson’s disease may not solely result from dopaminergic mechanisms but could also be associated with broader brain atrophy.^55^ Consequently, these changes might be less responsive to external cues, such as those provided in behavioural speech intervention. Furthermore, the participants in this cohort of people with Parkinson’s disease presented with mild symptoms on speech articulation even pre-intervention. The acoustic measures that are validated to reflect articulatory deficits in hypokinetic dysarthria in Parkinson’s disease are sparse. Measures of vowel articulation have been suggested to potentially differentiate between hypokinetic dysarthria and healthy speech, and effects on vowel space area following LSVT-LOUD® have been shown.^24,56^ However, the Vowel Articulation Index has been suggested to be a more stable, reliable and sensitive measure of vowel articulation than for example the vowel space area.^57^ Although we found no changes post-HiCommunication, it is of interest to evaluate intervention effects on articulation using other measures.

#### Six-month follow-up

The improvements in the acoustic measures of voice sound level and voice quality observed post-intervention compared to pre-intervention were not retained at the follow-up after six months. Of note, the dropout at follow-up was large, reducing the power to detect potential differences. However, it is reasonable to anticipate a loss of intervention effects and/or a progression of disease symptoms. Due to the progressive nature of Parkinson’s disease and its fluctuating symptomology, ongoing management is generally necessary. Studies of long-term effects of the active control intervention, HiBalance, have shown that intervention effects diminish after 6–12 months.^58,59^ Furthermore, the maintenance of improved speech and communication abilities is a challenge for many individuals. Although improvements in vocal loudness may persist to some degree following e.g. LSVT-LOUD®, life-long maintenance through home exercises and repeated intervention sessions are generally recommended.^10^ The results from our study further suggest that interventions in Parkinson’s disease, such as HiCommunication, may benefit from frequent repetition to uphold their positive effects.

### Speech-voice network changed post-HiCommunication

We found significant effective connectivity increases and decreases in a speech-motor-related network that was based on a recent LSVT-LOUD® study in people with Parkinson’s disease.^15^ Post-HiCommunication, there was a slightly decreased left supplementary motor area self-connectivity and an increased right supplementary motor area to left paracentral gyrus connectivity compared to the active controls (group-by-time effect). Comparing only the HiCommunication group pre- versus post-intervention, increased self-connectivity in the right supplementary motor area as well as between the left paracentral gyrus and left supplementary motor area were found. Alterations in the supplementary motor area were hypothesized since it plays a role in vocalization and initiation, articulation and control of speech-motor production.^28,60-62^ Baumann *et al*.^15^ found a partial normalization of supplementary motor area hypoactivation after LSVT-LOUD® in people with Parkinson’s disease. This is in line with our results showing that post-HiCommunication, connectivity was higher than before with and within the supplementary motor area that might hint towards a normalization of connectivity. Further, self-connectivity changes are a sign of adaptation of neuronal populations to local influence.^63^ In contrast, in a study measuring changes in regional cerebral blood flow using positron emission tomography, people with Parkinson’s disease had reduced activity in the supplementary motor area, among other areas, during an overt speech-motor task after LSVT-LOUD®.^15,16^

Even though HiCommunication altered effective connectivity as well as speech and voice outcomes, the changes were not significantly associated in parametric empirical Bayes association analyses. An argument could be made that the ROIs were taken from a task-based fMRI study, an activation that is directly related to a speech-motor task. In the present study, rsfMRI—a task-free measure of intrinsic connectivity—was used. Thus, connectivity changes in and between the ROIs may not be directly related to solely one acoustic measure. Additionally, we cannot be certain that the acoustic measures used as speech and voice outcomes in this study capture all improvements in the speech-motor-related network. However, the results may indicate that HiCommunication leads to more general alterations of the speech-motor-related network. This would not be surprising since we found several statistically significant changes in speech and voice measures post-intervention. The combination of these measures may be driving the network changes. To the best of our knowledge, there is no other rsfMRI study that could be discussed in the light of our data. Hence, comparability to prior studies is limited.

### Limitations

Intention-to-treat and per-protocol analyses of the speech and voice outcomes generated similar results. An important note is that analysis of complete cases may suffer more chance variation, and that under the missing at random assumption multiple imputation should correct biases that may arise in per-protocol analyses.^64^ However, there was a rather large amount of missing data post-intervention. Since imputation models are less reliable when the amount of missing data is larger, the results should be somewhat cautiously interpreted. Since the amount of missing data at follow-up (six months) was too large to enable reliable intention-to-treat analyses, we performed solely per-protocol follow-up analyses. The number of participants was rather low (*n* = 43), potentially reducing the power to detect any true differences. Furthermore, for the rsfMRI analysis, a low power (*n* = 26) could be a reason why the association analyses were non-significant. We based our rsfMRI hypothesis regarding the choice of our ROI and network on an fMRI study using a similar intervention. As abovementioned, the choice of ROI is crucial, and other ROI as well as another network or study as reference might have led to different results. In general, the rsfMRI analysis method heavily influences the results and is a large limitation regarding the reproducibility and stability of the results. See Supplement for discussion on limitations of methods for acoustic analysis.

## Conclusions

We showed clinically relevant improvements in voice sound level post-HiCommunication. Furthermore, there were group mean changes as well as trends of reliable changes in measures of voice quality. Changes in effective connectivity were found post-HiCommunication, including local neuronal adaptations. Nevertheless, there were no direct associations with speech and voice measures. In future studies, we will further evaluate speech outcomes including voice quality using audio-perceptual and self-reported measures. The consecutive step in the evaluation of HiCommunication is to investigate barriers and facilitators for implementing the intervention in different health care settings.

## Supplementary Material

fcae235_Supplementary_Data

## Data Availability

Data were collected within the framework of an RCT, the EXPANd trial.^13^ The analysis scripts and meta-data are available on the EXPANd OSF page (https://osf.io/s952g/). The study was described according to the CONSORT guidelines for reporting randomized controlled trials (http://www.consort-statement.org/). The original data are not publicly available due to Swedish/EU personal data legislation. Upon a reasonable request, data sharing will be regulated via a data transfer and user agreement.
