# Supplementary material for: Speech and neuroimaging effects following HiCommunication: a randomized controlled group intervention trial in Parkinson’s disease
Source: Brain Commun. 2024 Jul 12;6(4):fcae235. doi: 10.1093/braincomms/fcae235 (PMC11267236; doi:10.1093/braincomms/fcae235)
Supplement: fcae235_Supplementary_Data [file fcae235_supplementary_data.pdf]

Supplementary information to:

Speech and neuroimaging effects following HiCommunication: a randomised controlled group intervention trial in Parkinson's disease

Table of contents

Speech and neuroimaging effects following HiCommunication: a randomised controlled group intervention trial in Parkinson's disease 1

Table of contents 1

Adherence and missing data 2

Acoustic outcome variables and analysis procedures 3

Voice function 3

Voice sound level in text reading, monologue, and noise 3

Maximum Phonation Time 3

Voice quality 3

Acoustic Voice Quality Index and Harmonics-to-noise ratio 4

Prosody 4

Fundamental Frequency standard deviation 4

Articulation 4

Diadochokinesis sequential motion rate and alternating motion rate 5

Vowel Articulation Index 5

Deviations from preregistration 6

Methodological discussion 6

Supplementary Tables 7

Intervention 7

Supplementary Table 1. Description of structure, content, progression and core areas of the HiCommunication intervention. 7

Intrarater reliability 8

Supplementary Table 2. Results of ICC using single-rating, absolute-agreement, 2-way random-effects model 8

Predictors for multiple imputation 9

Supplementary Table 3. Correlation matrix of the demographic and outcome variables 9

Per-protocol analysis 11

Supplementary Table 4. Estimates of the per-protocol analysis (n=74) 11

Follow-up analysis 12

Supplementary Table *5*. Estimates of the follow up analysis (n=43) 12

Supplementary references 14

Adherence and missing data

Acoustic pre- or post-data were missing due to defective speech recordings for some participants. In the HiCommunication group, one participant was missing voice sound level (text reading, monologue and noise) pre-intervention, one was missing voice sound level in noise post-intervention and one was missing Vowel Articulation Index (VAI) pre- and post-intervention. In the active control group one participant was missing all acoustic data post-intervention, one was missing voice sound level (text reading, monologue and noise) pre-intervention, one was missing voice sound level in monologue post-intervention, one was missing F0 variability post-intervention and two were missing VAI pre- and post-intervention.

Six-month follow-up acoustic data were available for 20 participants (43%) in the HiCommunication group and 23 (48 %) in the active control group. Thus, only per-protocol follow-up analyses were performed. Out of those participants, some were missing one or more of the acoustic variables. In the HiCommunication group, one was missing voice sound level in monologue, F0 variability, VAI, Acoustic Voice Quality Index (AVQI) and Harmonics-to-Noise Ratio (HNR), one was missing voice sound level (text reading, monologue and noise) and one was missing VAI. In the active control group, two were missing voice sound level in monologue, F0 variability, VAI, AVQI and HNR, one was missing AVQI and HNR and one was missing VAI.

Out of the 28 HiCommunication participants with pre- and post- resting-state functional MRI (rsfMRI), one had missing pre-intervention data on voice sound level and was excluded. Due to missing Levodopa-equivalent daily dosage (LEDD) data, we excluded one further HiCommunication participant for the hierarchical model. Out of the 32 active controls, two had missing LEDD data and were excluded.

Acoustic outcome variables and analysis procedures

Voice function

The averaged speech loudness within a predefined time segment (text reading, monologue, or text reading in noise) and speech breathing (the maximum phonation time of a sustained vowel) were chosen to represent the speech domain voice function.

Voice sound level in text reading, monologue, and noise

Analysis was performed using the software Sopran (version 1.0.22 © Tolvan Data). For analysis of text reading the entire text without the title was used. For analysis of monologue, a 30 second interval from the mid portion of the monologue was used. In cases where the monologue was < 30 seconds, the entire monologue was used. For analysis of voice sound level in noise, the initial 215 syllables of the text were used. Participants read the text aloud whilst pink noise (70-72 decibel (dB)) was played in headphones (Sony MDR-ZX660AP). To reduce the impact of low-frequency background noise on the sound level, a C-weighted decibel (dBC) was used to report the voice sound level for all measures.

Maximum Phonation Time

Analysis was performed using the software Sopran (version 1.0.22 © Tolvan Data). The spectrogram was visually inspected to ensure that stable phonation was analysed. In cases where several repetitions of the sustained vowel were recorded, the best (i.e., longest) attempt was used for analysis.

Voice quality

The Acoustic Voice Quality Index (AVQI) and the Harmonics-to-noise ratio (HNR) were chosen to represent the speech domain voice quality. The AVQI (version 01.03, Phonanium, 2021) is a composite measure that combines several acoustic parameters to obtain a single score for the estimation of dysphonia [1,2]. The equation of the AVQI includes the smoothed cepstral peak prominence, Harmonics-to-Noise Ratio (HNR), shimmer local, shimmer local decibel (dB), general slope of the spectrum, and tilt of the regression line through the spectrum. The parameters are weighted together through linear regression analysis and converted to a score on a linear scale between 0–10. The limit for what is considered a dysphonic voice according to AVQI varies across languages. Since AVQI has not yet been

evaluated for Swedish, the limit validated for Dutch speakers was used (AVQI score = 2.95). Scores below the limit value are considered to represent a non-dysphonic voice quality. HNR is a measure of the proportion of harmonic sound to noise in the voice measured in decibels. HNR quantifies the relative amount of additive noise. The lower the HNR, the more noise in the voice[3].

Acoustic Voice Quality Index and Harmonics-to-noise ratio

The analysis tool AVQI (version 01.03, Phonanium, 2021) was used for AVQI analysis. The middle three seconds were extracted from a sustained vowel [a:], with a margin of 0.10 seconds. In the recordings which included repeated attempts at the sustained vowel, the last attempt was consistently used for analysis. In addition, extractions of a pre-chosen 45 syllables of text reading were analysed. HNR analysis was performed within the AVQI analysis in the same manner.

Prosody

Pitch (fundamental frequency (F0)) variability was chosen to represent the speech domain prosody. Pitch variability reflects the natural changes in voice pitch.

Fundamental Frequency standard deviation

Analysis was performed using the software Praat (version 6.0.36) [4]). The entire text without the title was used for analysis. Because F0 detection by taking default settings in Praat may be error-prone, a Praat script ("Get_speakers_register.praat") using an algorithm for automatic estimation of pitch floor and pitch ceiling was used[5].

Articulation

Measures of articulatory diadochokinesis (DDK) as well as of vowel articulation were chosen to represent the speech domain articulation. DDK measures are designed to estimate the rate and regularity of consonant-vowel syllable repetitions. Alternating motor rates (AMR) (measured in syllables per second) are regarded to reflect the motor abilities of speech articulators to reveal their movement limitations[6]. Sequential motor rates (repetition of the sequence /pa-ta-ka/) is generally more challenging to perform because of the alternation of bilabial, alveolar, and velar place of articulation. The DDK SMR rate has been shown to be

altered in participants with PD compared to healthy controls[6]. The normative median value for DDK AMR (/pa-pa-pa/) is 6.4 (SD 1.0) syllables/second and for DDK SMR 5.8 syllables/second (SD 1.0) for Swedish healthy adults [7]. Measures of vowel space, including Vowel Articulation Index (VAI), may capture a reduced articulatory range of motion in hypokinetic dysarthria [8]. The method makes it possible to get an overall picture of a person's articulation by only measuring the formant frequency values of the corner vowels /a/, /i/ and /u/. The VAI is a theoretically driven and empirically tested metric developed to represent vowel formant centralization, i.e., formants that normally have high center frequencies tend to have lower frequencies, and formants that normally have low center frequencies tend to have higher frequencies. The VAI has shown promise to more effectively reduce inter-speaker variability noise while maintaining high sensitivity to vowel centralization compared to the more traditional metric vowel space area (VSA)[8,9]. The VAI is expressed as:

$$(F2i + F1a) \div (F2u + F2a + F1u + F1i) \qquad (1)$$

Diadochokinesis sequential motion rate and alternating motion rate

Analysis was performed in Sopran (version 1.0.22 © Tolvan Data). Repetitions of the syllables /pa-pa-pa/ and /pa-ta-ka/, respectively, were visually inspected and a 5 second interval from a stable portion of the syllable repetition was used for analyses. In cases where 5 seconds of stable repetition was not available, the entire portion of stable repetition was used.

Vowel Articulation Index

Analysis was performed using the software Praat (version 6.0.36)). The aim was to extract 10 repetitions of each corner vowel from the speech material (sentences and text reading). However, since the speech material was not priorly adapted to facilitate VAI analysis there were sometimes less than 10 repetitions for each corner vowel. Consequently, to minimise drop-out a lower limit was set to 6 repetitions of each corner vowel. To obtain the formant frequency values, 30 milliseconds in the middle of each vowel were analysed and each formant was visually inspected to ensure that the 30 milliseconds was extracted from a stable part of the formant. Furthermore, outliers were reexamined to ensure that the incorrect formant had not been measured. The VAI was then calculated from the mean values of the formant frequency values extracted from each vowel using the formula (1).

Deviations from preregistration

We deviated from the protocol by using multiple imputation to handle missing data. Regarding the dynamic causal modelling (DCM) analyses, we additionally included LEDD as a regressor of no interest. We planned a paired t-test comparing pre- and post-intervention but decided to first perform an analysis of the group-by-time interaction effect using a hierarchical second-level model. We did not use the healthy control cohort for any rsfMRI comparisons. We did not include analysis of whether baseline characteristics of speech and voice predict intervention response.

Methodological discussion

Acoustic analysis is a widespread tool in clinics and research to analyse speech disorders and is often suitable for detection of hypokinetic dysarthria even at an early stage in the disease progression when symptoms may be relatively mild[10]. However, there are limited guidelines on which acoustic measures to use to specifically target disorders of relevant speech domains. We used guidelines developed in a study by Rusz and colleagues as one of four criteria for which acoustic measures to use as outcomes of HiCommunication[11]. To ensure that the acoustic outcomes are valid in terms of representing the speech dimensions associated with hypokinetic dysarthria as well as capturing the treatment effects post-HiCommunication, further studies in the project will investigate whether the acoustic outcomes are correlated to e.g., audio-perceptual measures of speech and voice.

Supplementary Tables

Intervention

Supplementary Table 1. Description of structure, content, progression and core areas of the HiCommunication intervention.

**HiCommunication**	**Core areas**	**Progression (blocks)**		
10 weeks		A: week 1-2	B: week 3-6	C: week 7-10
**Group training**<br>**2 sessions/week**	Voice intensity<br><br>Articulatory precision<br><br>Word retrieval<br><br>Memory	Exercises with focus on breathing, phonation and articulation. Establishing increased vocal loudness while maintaining good voice quality.	Increased level of difficulty of the exercises. Introducing memory games and associational tasks to increase cognitive load during exercises.	Complexity increased by increasing difficulty of memory games, incorporating more interaction between participants and adding background noise.
		Week 1-10		
**Home exercises with supporting training diary**<br>**1 session/week**		Relaxation and breathing exercises<br><br>Voice and speech exercises<br><br>Word and memory exercises		

Intrarater reliability

Supplementary Table 2. Results of ICC using single-rating, absolute-agreement, 2-way random-effects model

Outcome	ICC	95% CI LL	95% CI UL	F test value	df1	df2	p-value
Voice sound level text	1	1	1	0,446	39	39	7,73e-81
Voice sound level monologue	0,997	0,994	0,998	636	39	39	7,09e-45
Voice sound level noise	1	1	1	38575	38	38	1,28e-77
Sustained vowel duration	1	1	1	20668	39	39	2,48e-74
F0 variability	1	1	1	571269	39	39	1,92e-67
AVQI	0,974	0,951	0,986	75,8	39	39	4,71e-27
DDK AMR	0,959	0,924	0,978	47,9	39	39	2,79e-23
DDK SMR	0,966	0,938	0,982	58,6	39	39	6,16e-25
VAI	0,986	0,974	0,993	143	39	39	2,69e-33

ICC: Intraclass correlation coefficient. CI: Confidence interval. LL: lower limit. UL: upper limit. df: degrees of freedom. F0 variability: Fundamental frequency standard deviation in semitones (log transformed). AVQI: Acoustic Voice Quality Index, HNR: Harmonics to noise ratio, DDK-AMR and DDK-SMR: diadochokinetic rate alternating and sequential motion rates, VAI: Vowel Articulation Index

Predictors for multiple imputation

Supplementary Table 3. Correlation matrix of the demographic and outcome variables

Outcome	1	2	3	4	5	6	7	8	9	10	11	12	13	14	15	16	17	18	19
**1. Sustained vowel duration**	1	0.26	-0.01	-0.08	0.09	0.02	0.29	-0.16	-0.09	0.08	-0.21	0.09	0	-0.18	-0.17	-0.15	-0.01	-0.24	-0.15
**2. DDK AMR**	0.26	1	0.48	-0.18	0.29	0.27	0.54	-0.05	0.02	0.08	-0.4	0.04	0.11	-0.29	-0.18	-0.12	0.21	-0.51	-0.03
**3. DDK SMR**	-0.01	0.48	1	0.02	0.09	0.07	0.22	-0.06	-0.02	0.21	-0.14	-0.06	0.2	-0.04	-0.11	-0.03	-0.03	-0.18	0.24
**4. F0 variability**	-0.08	-0.18	0.02	1	0.07	0.12	-0.03	0.05	-0.19	-0.06	0.32	0.05	0.19	-0.13	0.02	-0.01	0	0.01	0.1
**5. Voice sound level text**	0.09	0.29	0.09	0.07	1	0.88	0.64	-0.17	0	0.03	-0.23	0.11	0.01	-0.02	-0.05	0.08	-0.01	-0.12	0.12
**6. Voice sound level monologue**	0.02	0.27	0.07	0.12	0.88	1	0.59	-0.14	0.02	0.1	-0.14	0.04	-0.02	-0.05	-0.07	0.01	0.01	-0.1	0.13
**7. Voice sound level noise**	0.29	0.54	0.22	-0.03	0.64	0.59	1	-0.07	-0.03	0.11	-0.27	0.05	0.05	-0.21	-0.13	-0.08	0.1	-0.24	-0.05
**8. AVQI**	-0.16	-0.05	-0.06	0.05	-0.17	-0.14	-0.07	1	-0.62	0.19	-0.46	0.12	-0.15	0.01	0.17	0.08	-0.14	0.11	-0.04
**9. HNR**	-0.09	0.02	-0.02	-0.19	0	0.02	-0.03	-0.62	1	-0.12	0.21	-0.11	-0.03	-0.02	-0.14	-0.1	0.13	-0.11	0.04
**10. VAI**	0.08	0.08	0.21	-0.06	0.03	0.1	0.11	0.19	-0.12	1	-0.23	0.03	-0.02	-0.01	-0.16	-0.16	-0.17	-0.2	0.12
**11. Sex**	-0.21	-0.4	-0.14	0.32	-0.23	-0.14	-0.27	-0.46	0.21	-0.23	1	-0.07	0.24	0.02	-0.07	-0.11	0.04	0.09	0.01
**12. Age**	0.09	0.04	-0.06	0.05	0.11	0.04	0.05	0.12	-0.11	0.03	-0.07	1	-0.15	-0.05	0.12	0.05	-0.22	-0.02	-0.06
**13. MoCA**	0	0.11	0.2	0.19	0.01	-0.02	0.05	-0.15	-0.03	-0.02	0.24	-0.15	1	-0.02	-0.12	-0.06	0.07	-0.2	0.13
**14. PDQ39**	-0.18	-0.29	-0.04	-0.13	-0.02	-0.05	-0.21	0.01	-0.02	-0.01	0.02	-0.05	-0.02	1	0.23	0.5	-0.12	0.36	0.27

Outcome	1	2	3	4	5	6	7	8	9	10	11	12	13	14	15	16	17	18	19
**15. UPDRS 3**	-0.17	-0.18	-0.11	0.02	-0.05	-0.07	-0.13	0.17	-0.14	-0.16	-0.07	0.12	-0.12	0.23	1	0.86	-0.02	0.39	0.07
**16. UPDRS**	-0.15	-0.12	-0.03	-0.01	0.08	0.01	-0.08	0.08	-0.1	-0.16	-0.11	0.05	-0.06	0.5	0.86	1	-0.02	0.35	0.16
**17. Presence intervention**	-0.01	0.21	-0.03	0	-0.01	0.01	0.1	-0.14	0.13	-0.17	0.04	-0.22	0.07	-0.12	-0.02	-0.02	1	-0.28	-0.17
**18. Dysarthria test score**	-0.24	-0.51	-0.18	0.01	-0.12	-0.1	-0.24	0.11	-0.11	-0.2	0.09	-0.02	-0.2	0.36	0.39	0.35	-0.28	1	0.13
**19. LEDD**	-0.15	-0.03	0.24	0.1	0.12	0.13	-0.05	-0.04	0.04	0.12	0.01	-0.06	0.13	0.27	0.07	0.16	-0.17	0.13	1

The numbers in the first row correspond to the numbered outcomes in the first column. All values are Pearson's correlation coefficients. F0 variability: Fundamental frequency standard deviation in semitones (log transformed). AVQI:, Acoustic Voice Quality Index, HNR: Harmonics-to-noise ratio. DDK-AMR and DDK-SM: diadochokinetic rate alternating and sequential motion rates. VAI: Vowel Articulation Index. MDS-UPDRS Movement Disorders Society – Unified Parkinson's Disease Rating Scale. PDQ-39: the Parkinson's Disease Questionnaire-39. MoCA: Montreal Cognitive Assessment, higher scores reflect a higher level of global cognitive function. LEDD: Levodopa Eqivalent Daily Dose

Per-protocol analysis

Supplementary Table 4. Estimates of the per-protocol analysis (n=74)

		group		time		group:time interaction		intercept	
	reg	b (95% CI)	*p*	b (95% CI)	*p*	b (95% CI)	*p*	b (95% CI)	*p*
Voice sound level text reading*	lin.	0.2	0.838	-0.1	0.885	2.1	**0.002**	70.8	< 0.0001
-	-	(-1.6, 2)	-	(-0.9, 0.8)	-	(0.8, 3.3)	-	(69.6, 72.1)	-
Voice sound level monologue	lin.	-0.1	0.907	-0.3	0.572	2.1	**0.004**	69.2	< 0.0001
-	-	(-2, 1.8)	-	(-1.3, 0.7)	-	(0.7, 3.5)	-	(67.9, 70.5)	-
Voice sound level noise	lin.	-0.6	0.586	-0.2	0.771	1.1	0.176	80.1	< 0.0001
-	-	(-2.7, 1.5)	-	(-1.2, 0.9)	-	(-0.5, 2.7)	-	(78.6, 81.5)	-
Sustained vowel duration	lin	1.6	0.324	-1.3	0.232	0.5	0.759	18.4	< 0.0001
-	-	(-1.6, 4.9)	-	(-3.4, 0.8)	-	(-2.6, 3.5)	-	(16.1, 20.6)	-
F0 variaiblity	log lin.	-0.2	**0.047**	0	0.325	0.1	0.116	1.1	< 0.0001
-	-	(-0.3, 0)	-	(-0.1, 0)	-	(0, 0.2)	-	(1, 1.2)	-
AVQI	lin.	0	0.89	0	0.916	-0.5	**0.031**	4.1	< 0.0001
-	-	(-0.5, 0.4)	-	(-0.3, 0.3)	-	(-0.9, -0.1)	-	(3.8, 4.4)	-
HNR	lin.	-0.6	0.274	0	0.907	1.3	**0.013**	16.9	< 0.0001
-	-	(-1.8, 0.5)	-	(-0.7, 0.8)	-	(0.3, 2.4)	-	(16.1, 17.7)	-
DDK AMR	lin.	0	0.975	-0.1	0.307	0.2	0.281	6.1	< 0.0001
-	-	(-0.4, 0.4)	-	(-0.3, 0.1)	-	(-0.1, 0.5)	-	(5.9, 6.4)	-
DDK SMR	lin	-0.1	0.561	0	0.953	0.3	0.088	5.4	< 0.0001
-	-	(-0.6, 0.3)	-	(-0.3, 0.3)	-	(0, 0.7)	-	(5.1, 5.7)	-
VAI	lin.	0	0.054	0	0.081	0	0.464	1	< 0.0001
-	-	(-0.1, 0)	-	(0, 0)	-	(0, 0)	-	(1, 1.1)	-

The column Reg. defines the type of multilevel model (mlm) used for the outcome; lin. = linear, log lin. = linear mlm on logged values, b = unstandardised estimate. CI: Confidence interval. LL: lower limit. UL: upper limit. F0 variability: Fundamental frequency standard deviation in semitones (log transformed), higher values indicate a more varied pitch. AVQI: Acoustic Voice Quality Index, scores above 2.95 indicate dysphonia, HNR: Harmonics to noise ratio, higher values indicate stronger tonal components compared to noise in the signal, DDK-AMR and DDK-SMR: diadochokinetic rate alternating and sequential motion rates in syllables per second, VAI: Vowel Articulation Index; higher values indicate better articulatory ability and clarity in vowel production.

Follow-up analysis

Supplementary Table 5. Estimates of the follow up analysis (n=43)

		group		time 1		time 2		group:time 1 interaction		group:time 1 interaction		intercept	
	**Reg**	**b (95% CI)**	***p***	**b (95% CI)**	***p***	**b (95% CI)**	***p***	**b (95% CI)**	***p***	**b (95% CI)**	***p***	**b (95% CI)**	***p***
Voice sound level text reading	lin.	0.5	0.598	0	0.964	0.1	0.909	1.8	0.034	1.6	0.158	70.8	< 0.0001
-	-	(-1.5, 2.6)	-	(-1.2, 1.1)	-	(-1.3, 1.5)	-	(0.2, 3.5)	-	(-0.6, 3.7)	-	(69.4, 72.2)	-
Voice sound level monologue	lin.	0	0.991	-0.2	0.758	-0.8	0.345	1.8	0.049	1.8	0.142	69.3	< 0.0001
-	-	(-2.1, 2.1)	-	(-1.4, 1)	-	(-2.4, 0.8)	-	(0, 3.6)	-	(-0.6, 4.2)	-	(67.9, 70.7)	-
Voice sound level noise	lin.	0	0.996	0.2	0.756	-1.5	0.058	0.2	0.793	2.3	0.057	79.9	< 0.0001
-	-	(-2.4, 2.4)	-	(-1, 1.4)	-	(-3.1, 0)	-	(-1.6, 2.1)	-	(0, 4.6)	-	(78.3, 81.5)	-
Sustained vowel duration	lin	1.9	0.3	-1.8	0.093	-1.4	0.295	1.4	0.378	0.9	0.66	18.7	< 0.0001
-	-	(-1.7, 5.6)	-	(-3.9, 0.3)	-	(-4.1, 1.2)	-	(-1.7, 4.5)	-	(-3, 4.8)	-	(16.3, 21.2)	-
F0 sd	log lin.	-0.2	0.046	-0.1	0.155	-0.1	0.189	0.1	0.088	0.2	**0.045**	1.1	< 0.0001
-	-	(-0.3, 0)	-	(-0.1, 0)	-	(-0.2, 0)	-	(0, 0.2)	-	(0, 0.3)	-	(1, 1.2)	-
AVQI	lin.	0	0.897	0	0.92	-0.1	0.617	-0.5	0.025	-0.4	0.157	4.1	< 0.0001

-	-	(-0.5, 0.4)	-	(-0.3, 0.3)	-	(-0.5, 0.3)	-	(-0.9, -0.1)	-	(-1, 0.2)	-	(3.8, 4.4)	-
HNR	lin.	-0.9	0.131	-0.1	0.781	0	0.922	1.4	0.01	1.2	0.103	17.1	< 0.0001
-	-	(-2.1, 0.3)	-	(-0.8, 0.6)	-	(-1, 0.9)	-	(0.4, 2.5)	-	(-0.2, 2.6)	-	(16.3, 17.9)	-
DDK papapa	lin.	0.1	0.709	-0.1	0.676	-0.4	0.005	0.1	0.485	0.3	0.153	6.1	< 0.0001
-	-	(-0.4, 0.5)	-	(-0.3, 0.2)	-	(-0.7, -0.1)	-	(-0.2, 0.5)	-	(-0.1, 0.8)	-	(5.8, 6.4)	-
DDK pataka	lin	-0.1	0.683	0	0.905	0	0.934	0.3	0.174	0	0.943	5.3	< 0.0001
-	-	(-0.6, 0.4)	-	(-0.3, 0.3)	-	(-0.3, 0.4)	-	(-0.1, 0.7)	-	(-0.6, 0.5)	-	(5, 5.7)	-
VAI	lin.	0	0.031	0	0.079	0	0.789	0	0.266	0	0.574	1	< 0.0001
-	-	(-0.1, 0)	-	(0, 0)	-	(0, 0)	-	(0, 0)	-	(0, 0)	-	(1, 1.1)	-

The column Reg. defines the type of multilevel model (mlm) used for the outcome; lin. = linear, log lin. = linear mlm on logged values, b = unstandardised estimate. Time 1: Pre- to post-intervention. Time 2: pre-intervention to follow-up. CI: Confidence interval. LL: lower limit. UL: upper limit. F0 variability: Fundamental frequency standard deviation in semitones (log transformed), higher values indicate a more varied pitch. AVQI: Acoustic Voice Quality Index, scores above 2.95 indicate dysphonia, HNR: Harmonics to noise ratio, higher values indicate stronger tonal components compared to noise in the signal, DDK-AMR and DDK-SMR: diadochokinetic rate alternating and sequential motion rates in syllables per second, VAI: Vowel Articulation Index; higher values indicate better articulatory ability and clarity in vowel production.

Supplementary references

1. Maryn Y, De Bodt M, Roy N. The Acoustic Voice Quality Index: Toward improved treatment outcomes assessment in voice disorders. *Journal of communication disorders*. 2009;43(3):161-174.
2. Uloza V, Latoszek BBV, Ulozaite-Staniene N, Petrauskas T, Maryn Y. A comparison of Dysphonia Severity Index and Acoustic Voice Quality Index measures in differentiating normal and dysphonic voices. *European archives of oto-rhino-laryngology : official journal of the European Federation of Oto-Rhino-Laryngological Societies (EUFOS) : affiliated with the German Society for Oto-Rhino-Laryngology - Head and Neck Surgery*. 275(4):949-958.
3. Ferrand CT. Harmonics-to-noise ratio: an index of vocal aging. *J Voice*. 2002;16(4):480-7.
4. Boersma P, Weenik D. PRAAT: a system for doing phonetics by computer. *Glot international*. 2001:5:341-345.
5. De Looze C, Hirst D. Detecting changes in key and range for the automatic modelling and coding of intonation. In: *Proceedings of the 4th International Conference on Speech Prosody;* 2008;135-138.
6. Rusz J, Tykalova T, Novotny M, et al. Defining Speech Subtypes in De Novo Parkinson Disease: Response to Long-term Levodopa Therapy. *Neurology*. 2021;97(21):e2124-e2135.
7. Hartelius L. *Dysartri - bedömning och intervention : vid förvärvade neurologiska talstörningar hos vuxna*. Studentlitteratur; 2015.
8. Caverlé MWJ, Vogel AP. Stability, reliability, and sensitivity of acoustic measures of vowel space: A comparison of vowel space area, formant centralization ratio, and vowel articulation index. *J Acoust Soc Am*. 2020;148(3):1436.
9. Sapir S, Spielman JL, Ramig LO, Story BH, Fox C. Effects of Intensive Voice Treatment (the Lee Silverman Voice Treatment [LSVT]) on Vowel Articulation in Dysarthric Individuals With Idiopathic Parkinson Disease: Acoustic and Perceptual Findings. *Journal of Speech, Language, and Hearing Research*. 2007;50(4):899-912.
10. Brabenec L, Mekyska J, Galaz Z, Rektorova I. Speech disorders in Parkinson's disease: early diagnostics and effects of medication and brain stimulation. *Journal of Neural Transmission*. 2017;124(3):303-334.
11. Rusz J, Tykalova T, Ramig LO, Tripoliti E. Guidelines for Speech Recording and Acoustic Analyses in Dysarthrias of Movement Disorders. *Movement disorders*. 2021;36(4):803-814.
